# Integrated Transcriptomic and Proteomic Analyses of the Interaction Between Chicken Synovial Fibroblasts and *Mycoplasma synoviae*

**DOI:** 10.3389/fmicb.2020.00576

**Published:** 2020-04-03

**Authors:** Rui Liu, Bin Xu, Shengqing Yu, Jingfeng Zhang, Huawei Sun, Chuanmin Liu, Fengying Lu, Qunxing Pan, Xiaofei Zhang

**Affiliations:** ^1^Key Laboratory of Veterinary Biological Engineering and Technology, Ministry of Agriculture, Institute of Veterinary Medicine, Jiangsu Academy of Agricultural Sciences, Nanjing, China; ^2^National Center for Engineering Research of Veterinary Bio-products, Jiangsu Academy of Agricultural Sciences, Nanjing, China; ^3^Shanghai Veterinary Research Institute, Chinese Academy of Agricultural Sciences, Shanghai, China

**Keywords:** correlation analysis, chicken synovial fibroblasts, *Mycoplasma synoviae*, RNA-Seq, TMT

## Abstract

*Mycoplasma synoviae* (MS), which causes respiratory disease, eggshell apex abnormalities, infectious synovitis, and arthritis in avian species, has become an economically detrimental poultry pathogen in recent years. In China, the disease is characterized by infectious synovitis and arthritis. However, the mechanism by which MS causes infectious synovitis and arthritis remains unknown. Increasing evidence suggests that synovial fibroblasts (SF) play a key role in the pathogenesis of arthritis. Here, both RNA sequencing and tandem mass tag analyses are utilized to compare the response of primary chicken SF (CSF) following infection with and without MS. The host response between non-infected and infected cells was remarkably different at both the mRNA and protein levels. In total, 2,347 differentially expressed genes (DEGs) (upregulated, *n* = 1,137; downregulated, *n* = 1,210) and 221 differentially expressed proteins (DEPs) (upregulated, *n* = 129; downregulated, *n* = 92) were detected in the infected group. A correlation analysis indicated a moderate positive correlation between the mRNA and protein level changes in MS-infected CSF. At both the transcriptomic and proteomic levels, 149 DEGs were identified; 88 genes were upregulated and 61 genes were downregulated in CSF. Additionally, part of these regulated genes and their protein products were grouped into seven categories: proliferation-related and apoptosis-related factors, inflammatory mediators, proangiogenic factors, antiangiogenic factors, matrix metalloproteinases, and other arthritis-related proteins. These proteins may be involved in the pathogenesis of MS-induced arthritis in chickens. To our knowledge, this is the first integrated analysis on the mechanism of CSF-MS interactions that combined transcriptomic and proteomic technologies. In this study, many key candidate genes and their protein products related to MS-induced infectious synovitis and arthritis were identified.

## Introduction

*Mycoplasma synoviae* (MS) is a common poultry and extracellular pathogen that leads to acute or chronic respiratory diseases, infectious synovitis, and arthritis in avian species (Fletcher et al., 1976; Catania et al., 2016b; Michiels et al., 2016; Sun et al., 2017), and eggshell apex abnormalities in chickens (Feberwee et al., 2009; Catania et al., 2010). Control of MS infections generally involves eradication of the pathogen from breeder flocks, antibiotic usage, improvements in housing conditions, and vaccination with MS-H, a temperature-sensitive MS strain that is widely used in many countries (Gerchman et al., 2008). However, subacute and chronic infections make the control and elimination of this pathogen particularly difficult. For example, in China, the disease has resulted in the loss of millions of chickens in many regions and has negatively affected the economy of the poultry industry from 2010 to 2015 (Sun et al., 2017).

The clinical characteristics of MS infection are well known (Catania et al., 2016a; Sun et al., 2017; Ball et al., 2018; Kordafshari et al., 2019; Lorenc et al., 2019; Wu et al., 2019), and a number of genomic, proteomic, phenotype microarrays, and other analyses have been conducted. However, only a few MS proteins, including variable lipoprotein hemagglutinin (Narat et al., 1998; Lavric et al., 2007), cysteine protease (Cizelj et al., 2011), neuraminidase (Bercic et al., 2011), the putative nuclease MS53_0284 (Vasconcelos et al., 2005; Cizelj et al., 2016), and matrix metalloproteinase-2 (MMP-2) (Cizelj et al., 2016), have been identified as virulence determinants. In terms of host-pathogen interactions, Goret et al. (2017) showed that *M. hominis* lipoproteins played a role in the activation of human dendritic cells and their immune responses. Addis et al. (2011) utilized a proteomic approach to investigate the impact of *M. agalactiae* infection on mammary epithelium. Another study employed phenotype microarrays to investigate the influence of MS on the global metabolic activity of chicken chondrocytes (CCH) (Dusanic et al., 2014). A separate study performed a quantitative real-time polymerase chain reaction (qRT-PCR) assay of gene expression in an *in vitro* co-culture of MS and CCH (Cizelj et al., 2016). Nevertheless, the molecular pathogenesis of the disease is not well understood, and the nature of the host-pathogen interaction during MS infection has not been clarified. This lack of knowledge can be attributed to the absence of data on the effects of MS infection in host cell responses.

The aim of this study was to investigate the interaction between primary chicken synovial fibroblasts (CSF) and MS strain, HN01. RNA sequencing (RNA-Seq)-based transcriptomics and tandem mass tag (TMT)-based proteomics analyses were applied to identify differentially expressed genes (DEGs) and differentially expressed proteins (DEPs) in response to MS strain HN01 infection in CSF. Using obtained comprehensive quantitative datasets, key genes and their protein products involved in MS-induced arthritis were extracted.

## Materials and Methods

### MS Culture, Cell Culture, and Experimental Design

A wild-type MS strain, HN01, was isolated from broiler breeder flocks that exhibited clinical symptoms of MS from Henan Province. HN01 was grown in Frey mycoplasma broth, as previously described (Dusanic et al., 2009). The number of color-changing units (CCUs) was determined according to previously reported methods (Stemke and Robertson, 1982). Bacteria in the logarithmic phase of growth were used for cell infection.

Primary CSF were prepared from synovial tissues obtained from specific pathogen-free chickens. Synovial tissues were rinsed with sterile phosphate-buffered saline, minced into small pieces, dried with sterilization filter paper, and spread on the bottom of six-well cell plates in Dulbecco’s modified Eagle’s medium, supplemented with 7.5% fetal bovine serum, 2.5% chicken serum, 100 μg/mL streptomycin, and 100 U/mL penicillin at 37°C for 4–5 days (Supplementary Figure S1A). The culture medium was replaced every 3 days until primary cultures reached 90% confluence and subsequently recultivated. Cells at passages 3–6 were used in this study. To characterize the cytological phenotype of synovial cultures, cells were stained with anti-vimentin antibody (Boster Biological Technology Co. Ltd., Wuhan, China) (Hirohata et al., 2009). The majority of cells (98%) were positive for vimentin staining, which was measured by a Nikon N-STORM microscope (Nikon, Tokyo, Japan) (Supplementary Figure S1B).

CSF were plated on six-well culture dishes at a density of 3 × 10^5^ cells/well, and incubated overnight. After 24 h of serum starvation, cells were infected with MS at a MOI of 100 (5 × 10^7^ CCU/well) for 48 h. Control cells were mock stimulated with serum free medium for 48 h. Mock- and MS-infected CSF were collected from non-exposed and exposed CSF 48 h post-infection (hpi), respectively. Samples included three independent biological replicates.

### Total RNA Isolation and cDNA Library Construction

Total RNA was extracted from mock- and MS-infected CSF using Trizol (Invitrogen, CA, United States) following the manufacturer’s instructions. The integrity of total RNA was measured using a Nanodrop 2000 spectrophotometer and Agilent 2100 BioAnalyzer (Thermo Fisher Scientific, MA, United States). In a subsequent procedure, mRNA molecules were purified from total RNA using Oligo(dT)-attached magnetic beads. For quality control, the cDNA libraries were validated using an Agilent Technologies 2100 BioAnalyzer. Libraries were generated using the BGISEQ-500 platform (BGI, Shenzhen, China).

### RNA-Seq Data Analysis

Raw data were filtered using SOAPnuke v1.4.0 (Chen et al., 2018) and Trimmomatic v0.36 (Bolger et al., 2014) to obtain clean reads and remove low-quality reads with >5% unknown nucleotides, reads containing >20% low-quality bases with quality values < 10, and reads containing adaptor sequences. Clean reads were stored in FASTQ format and used for quantitative analysis.

All high-quality clean reads were mapped onto the reference genes that were available in the *Gallus gallus* reference genome GRCg6a^1^ using Bowtie2 v2.2.5 (Langmead and Salzberg, 2012). Matched reads were calculated at the gene expression level using RSEM software (Dewey and Li, 2011). For the analysis of DEGs, raw counts were utilized and compared across treatments using the DESeq2 method with a fold change (FC) ≥ 2 and adjusted *p*-value (*q*-value) ≤ 0.001 as the significance threshold (Wang et al., 2010).

### Protein Preparation, TMT Labeling, and Peptide Fractionation

Three biological replicates for each group were used for proteomic analyses. Samples were sonicated on ice in lysis buffer (8M urea, 1% protease inhibitors). After centrifugation at 12,000 *g* for 10 min at 4°C, the supernatant was transferred to a new centrifuge tube. Protein concentrations and qualities were determined using a Pierce^TM^ BCA Protein Assay kit (Thermo Fisher Scientific, MA, United States) and confirmed by SDS-PAGE. For digestion, the protein solution was treated with 5 mM dithiothreitol for 30 min at 56°C and alkylated with 11 mM iodoacetamide for 15 min at room temperature in the dark. Then, the sample was diluted to a urea concentration < 2 M by adding 100 mM triethyl ammonium bicarbonate (TEAB) (Sigma, St. Louis, MO, United States). For trypsin digestion, trypsin was added in a 50:1 protein-to-trypsin ratio overnight followed by a 100:1 ratio for 4 h. After trypsin digestion, the peptide was desalted using a Strata X C18 SPE column (Phenomenex, Torrance, United States) and vacuum dried. The peptide was reconstituted in 0.5 M TEAB and labeled by TMTs with a TMT labeling kit (Thermo Fisher Scientific, MA, United States) following the manufacturer’s instructions. In brief, one unit of TMT reagent was defrosted and restructured in acetonitrile. Then, the peptide mixture was incubated for 2 h at room temperature, pooled, desalted, and vacuum dried by centrifugation. Tryptic peptides were separated by high pH reversed-phase HPLC using an Agilent 300Extend C18 column (5 μm particles, 4.6 mm × 250 mm) (Agilent Technologies, CA, United States). Peptides were first separated with a gradient of 8–32% acetonitrile (pH 9.0) for 1 h into 60 fractions. Subsequently, peptides were merged into nine fractions and vacuum dried.

### Liquid Chromatography-Mass Spectrometry (LC-MS/MS) Analysis

Each fraction was resuspended in mobile phase A containing 0.1% formic acid and 2% acetonitrile. A linear gradient was formed as follows: an increase in solvent B (0.1% formic acid in 90% acetonitrile) from 9% to 25% for 30 min, an increase from 25% to 35% for 22 min, an increase to 80% for 4 min, and maintenance at 80% for the last 4 min at a constant flow rate of 350 nL/min on an EASY-nLC 1200 UPLC system (Thermo Fisher Scientific, MA, United States). Eluted peptides were subjected to a nanospray ionization source and analyzed by Oribitrap Fusion Lumos Mass Spectrometer (Thermo Fisher Scientific, MA, United States). A data-dependent manner that alternated between one mass spectrometry scan (60,000 resolving power) and 20 MS/MS (15,000 resolving power) scans with 30 s dynamic exclusion was performed.

### Protein Identification and Quantification Based on TMT Data

Mass spectrometry proteome data were deposited to the ProteomeXchange Consortium via the iProX partner repository (Ma et al., 2019). The resulting MS/MS data were analyzed using the MaxQuant search engine v.1.5.2.8 (Tyanova et al., 2016). Tandem mass spectra were searched against the *G. gallus* reference transcriptome (GRCg6a). Trypsin/P was specified as the cleavage enzyme, and two missed cleavages were permissible. Mass tolerance was set to 20 ppm for precursor ions of the first search, 5 ppm for precursor ions of the main search, and 0.02 Da for fragment ions. Carbamidomethyl on Cys was specified as fixed modifications, and oxidation on Met was specified as variable modifications. For protein quantification, TMT-6plex was employed and the false discovery rate was adjusted to <1%. Proteins with a FC > 1.3 and a *p*-value < 0.05 were considered differentially expressed.

### Statistical Analysis

To analyze biological replicates in the experimental groups and observe differences in the gene and protein expression changes between mock and infected groups, a principal component analysis (PCA) of the transcript and protein data was performed using the “princomp” function in R software package.

### Bioinformatics Analysis

For transcriptome data, DEGs were utilized for Gene Ontology (GO) and Kyoto Encyclopedia of Genes and Genomes (KEGG) enrichment analyses, which were further classified according to official classifications. Both GO terms and KEGG pathways with a *q*-value ≤ 0.05 were significantly enriched in DEGs according to the phyper function in the R software package.

For proteome data, DEPs were annotated with the GO and KEGG databases for functional analysis. For GO proteome annotation, the UniProt-GOA database^2^ was searched. For proteins not annotated in the UniProt-GOA database, InterProScan software^3^ was used to annotate the proteins’ GO function based on the protein sequence alignment method. Additionally, the KEGG online service tool, KAAS^4^, was used to annotate the proteins’ KEGG database description. To test the enriched DEPs against all identified proteins in the GO and KEGG pathways analyses, Fisher’s exact test (two-tailed) was conducted. The GO terms and KEGG pathways with a *p*-value < 0.05 were considered significant.

STRING database v.10.5^5^ was used to analyze the protein-protein interactions (PPIs) of selected proteins. An interaction network with a confidence score ≥ 0.7 (high confidence) was constructed, and the data were visualized using the R package, “networkD3.”

### Association and Co-expression Analyses

To investigate the potential relevance of quantitative information between mRNA and proteins, subsets of mRNA and proteins were screened using the following cut-off values: FC ≥ 2.0 and *q*-value ≤ 0.001 (DEGs); FC > 1.3 and *p*-value < 0.05 (DEPs). Then, RNA-Seq data were used as a searchable database. All identified protein sequences were analyzed and queried with the RNA-Seq data. Concordance between the transcriptome and proteome data was determined by Pearson’s correlation analysis with *R* > 0.80 denoting a significantly positive correlation and 0.50 < *R* < 0.80 denoting a moderate positive correlation.

### qRT-PCR

To confirm the RNA-Seq and TMT results, 19 DEGs were assessed using qRT-PCR. Reaction parameters were identical for all genes. Briefly, purified RNA was transformed into first-strand cDNA using PrimeScript^TM^ RT Master Mix (Takara, Japan) following the manufacturer’s instructions. qRT-PCR with cDNA and the appropriate minus RT controls was performed on the ABI StepOnePlus Real-Time PCR system (Applied Biosystems, Foster City, CA, United States) using Power SYBR Green PCR Master mix (Applied Biosystems, Foster City, CA, United States). In brief, after the initial reaction conditions at 50°C for 2 min and denaturation at 95°C, cDNA was amplified by 40 cycles of PCR (95°C for 15 s, 55°C for 15 s, 72°C for 1 min) on the ABI StepOnePlus Real-Time PCR system. Relative RNA expression levels were normalized using *actin* (Wang et al., 2018) and calculated using the 2^–ΔΔ*Ct*^ method (Schmittgen and Livak, 2008). All primers used in this study are provided (Supplementary Table S1).

## Results

### Transcriptomics Analysis of DEGs

**FIGURE 1:**
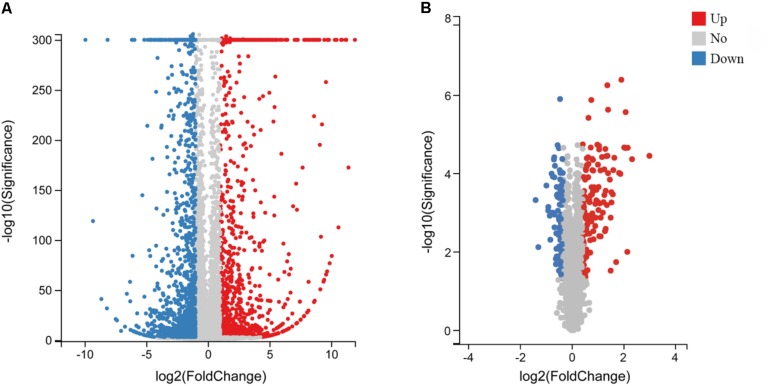
Volcano plot of DEGs and DEPs in MS-infected CSF. Volcano plot of DEGs **(A)** and DEPs **(B)**. Gray dots indicate genes or proteins without significant differential expression, red dots indicate significantly upregulated genes or proteins, and blue dots indicate significantly downregulated genes or proteins. Horizontal and vertical coordinates indicate the fold change differences and *q*-values of DEGs and DEPs, respectively.

To explore the molecular mechanisms involved in the response to MS infection and changes in gene expression, cDNA samples of CSF not exposed to MS infection and CSF 48 hpi were sequenced using the BGISEQ-500 platform. After sequencing and filtering for low-quality reads, clean reads were obtained. In total, 49.19–52.3 million raw sequence reads were generated from the mock and infected groups with percentages of total mapped reads and unique mapped reads ranging from 74.92 to 77.02 and 66.62 to 69.1, respectively (Supplementary Table S2). After mapping clean reads onto *G. gallus* genome, 15,400 genes were identified in the transcriptomics analysis (Supplementary Table S3). In response to infection, the expression levels of various host genes were remarkably altered. Compared to the untreated group and according to the high-throughput analysis, 2,347 DEGs (upregulated, *n* = 1,137; downregulated, *n* = 1,210) were identified in MS-infected CSF (Figure 1A and Supplementary Table S4).

### PCA of Transcriptome and Proteome Data

**FIGURE 2:**
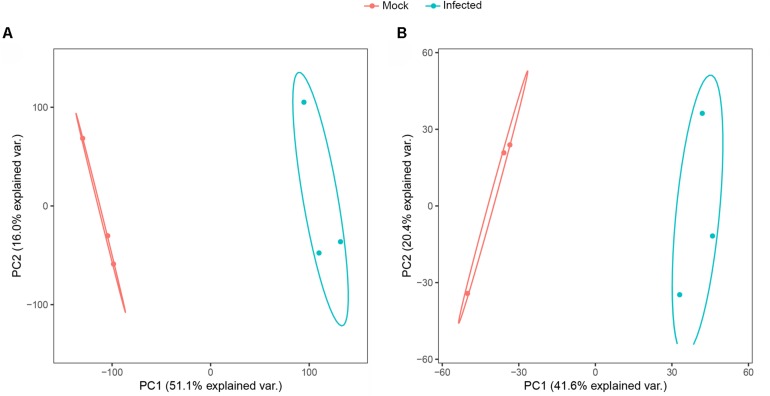
Principal component analysis score plot of transcriptome **(A)** and proteome **(B)** profiles.

PCA is a multivariate statistical analysis method that reduces multiple dimensions to a few independent variables while preserving as much of the original data as possible. PCA allows for grouping of samples with overall similar expression characteristics. And the higher the degree of aggregation between samples, the better the biological repeatability. Based on the PCA analysis, two experimental groups included six samples were clustered into 2 groups of a clear separation which indicated that there was a difference in the expression of genes and proteins between the experimental groups and the biological replicates were consistent in the transcriptomics and proteomics analyses (Figures 2A,B).

### GO and KEGG Pathway Enrichment Analyses of DEGs

**FIGURE 3:**
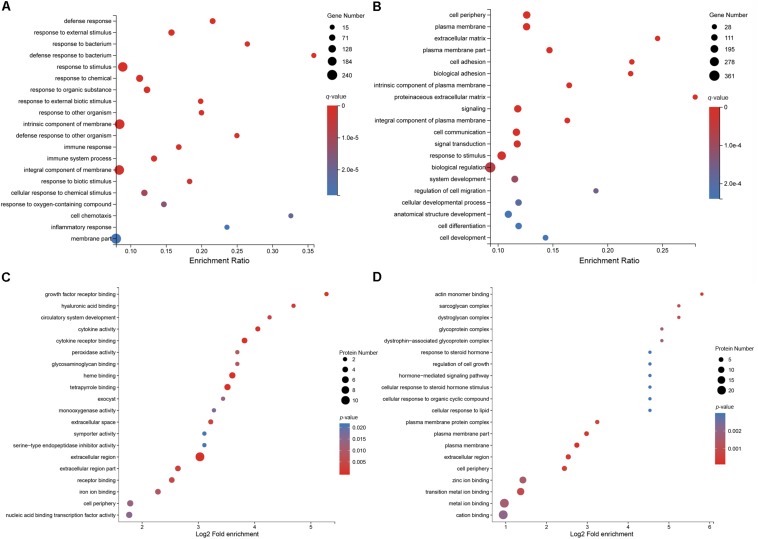
GO enrichment analysis of DEGs and DEPs in MS-treated CSF. Statistics of the top 20 enriched GO term for upregulated DEGs **(A)**, the top 20 enriched GO term for downregulated DEGs **(B)**, the top 20 enriched GO term for upregulated DEPs **(C)**, and the top 20 enriched GO term for downregulated DEPs **(D)**.

**FIGURE 4:**
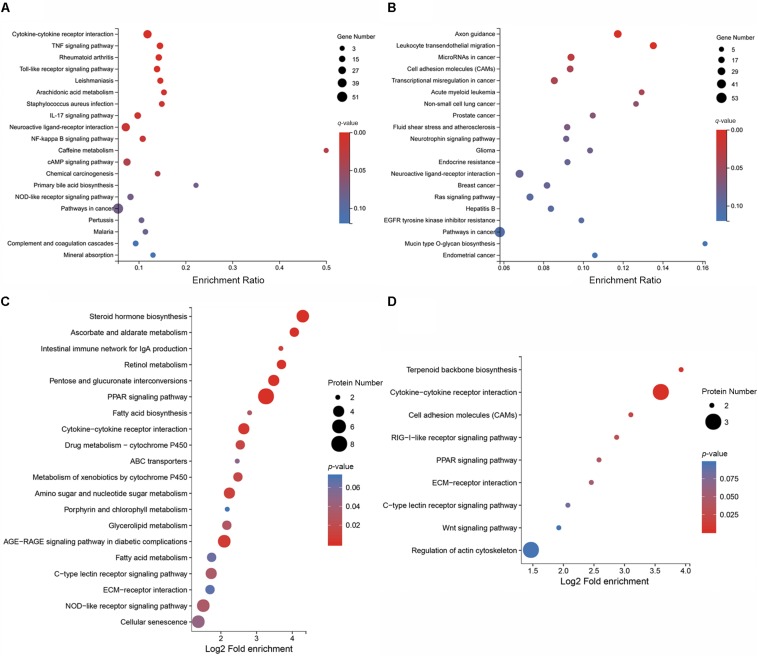
KEGG pathway enrichment analysis of DEGs and DEPs in CSF exposed to MS. KEGG pathway enrichment analysis of the top 20 enriched pathways for upregulated DEGs **(A)**, the top 20 enriched pathways for downregulated DEGs **(B)**, the top 20 enriched pathways for upregulated DEPs **(C)**, and the top 9 enriched pathways for downregulated DEPs **(D)**.

To elucidate the distribution of DEGs at the macro-level, each set of DEGs from the mock and treatment groups was mapped in accordance with the GO terms and KEGG pathways. The GO enrichment analysis revealed that upregulated genes in the infected group were strongly related to stimuli responses (i.e., defense, external stimulus/stimulus/chemical/organic substance/external biotic stimulus/biotic stimulus/oxygen-containing compound, immune response, and inflammatory response), multi-organism processes (i.e., response to bacteria or other organisms, including defense responses), cellular processes (i.e., cellular response to a chemical stimulus, including chemotaxis), membrane parts (i.e., intrinsic/integral components of the membrane or membrane parts), and immune system processes (Figure 3A). Most downregulated genes in MS-incubated CSF were associated with biological adhesion (i.e., cell/biological adhesion), cellular processes (i.e., cell communication/differentiation/development, cellular developmental processes, and cell), extracellular region (i.e., the extracellular matrix or proteinaceous extracellular matrix), biological regulation (i.e., signal transduction, biological regulation, or the regulation of cell migration), and cells (i.e., cell periphery, plasma membrane, plasma membrane part, and intrinsic/integral components of the plasma membrane) (Figure 3B). The KEGG pathway enrichment analysis demonstrated that most upregulated genes in the infected group were associated with environmental information processing (i.e., cytokine-cytokine receptor interactions, the TNF/NF-kappa B/cAMP signaling pathway, and neuroactive ligand-receptor interaction), metabolism (i.e., arachidonic acid and caffeine metabolism), and organismal systems (i.e., Toll-like/IL-17/NOD-like receptor signaling pathway, complement and coagulation cascades, and mineral absorption) (Figure 4A). Additionally, environmental information processing (i.e., cell adhesion molecules, neuroactive ligand-receptor interactions, and the Ras signaling pathway) and organismal systems (i.e., axon guidance, leukocyte transendothelial migration, and the neurotrophin signaling pathway) were the most enriched pathways among downregulated genes (Figure 4B).

### Proteomics Analysis of DEPs

To uncover the effects of MS infection on host gene expression at the translational level, TMT and LC-MS/MS analyses were performed. After quality validation, a total of 320,457 (82,753 matched) spectra were obtained. Of these spectra, 41,982 peptides (39,018 unique peptides) and 5,537 identified proteins (4,609 quantifiable proteins) were detected. Details on the protein mass spectrometry data are provided (Supplementary Table S5). In total, 221 DEPs were identified, of which, 129 proteins were upregulated and 92 proteins were downregulated with a FC > 1.3 and *p*-value < 0.05 (Figure 1B and Supplementary Table S6).

### GO and KEGG Pathway Enrichment Analyses of DEPs

To further characterize the functions of these DEPs, a GO enrichment analysis was conducted. The GO enrichment analysis revealed that the majority of upregulated DEPs in the MS-infected group were related to the extracellular region (i.e., extracellular region, extracellular region part, and extracellular space), cells (i.e., periphery, exocysts, cortex, cortex and its components, and the cytoplasmic region), and binding (i.e., heme, tetrapyrrole, cytokine receptor, and growth factor receptor binding) (Figure 3C). Most downregulated DEPs were preferentially associated with cells (i.e., the plasma membrane and its components, periphery, the plasma membrane protein complex, and the dystroglycan complex), binding (i.e., actin monomers, transition metal ions, zinc ions, metal ions, cations, and polysaccharide/pattern binding), cellular processes (i.e., cellular response to steroid hormones, organic cyclic compounds, and lipids), and cellular component organization or biogenesis (i.e., actin cytoskeleton and actin filament organization) (Figure 3D).

Functional classification of the DEPs was performed by mapping to the KEGG pathways using KAAS. Results revealed that upregulated proteins in MS-infected CSF were largely involved in metabolism (i.e., steroid hormone biosynthesis, pentose and glucuronate interconversions, ascorbate and aldarate metabolism, retinol metabolism, and amino sugar and nucleotide sugar metabolism), organismal systems (i.e., the PPAR signaling pathway, intestinal immune network for IgA production, NOD-like receptor signaling pathway, and C-type lectin receptor signaling pathway), and environment information processing (i.e., cytokine-cytokine receptor interactions) (Figure 4C). Among the downregulated proteins in MS-infected CSF, enriched pathways included cytokine–cytokine receptor interactions, terpenoid backbone biosynthesis, cell adhesion molecules, the RIG-I-like receptor signaling pathway, and the PPAR signaling pathway (Figure 4D).

### Correlation Analysis of the Transcriptome and Proteome Data in Mock and MS-Infected CSF

**FIGURE 5:**
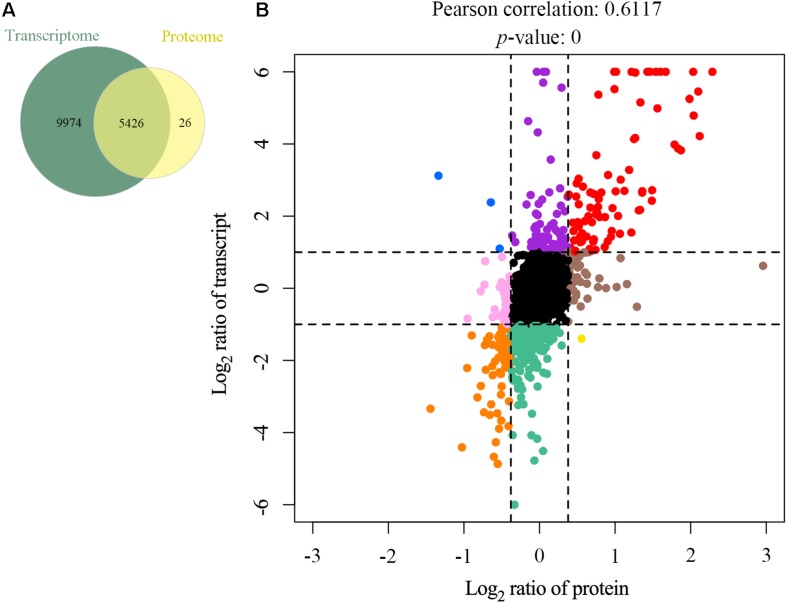
Relationship patterns of all quantitative mRNA and proteins. Comparison of the number of transcribed genes and expressed proteins **(A)**. Nine-quadrant diagram **(B)**. The abscissa represents log_2_ expression ratio from proteomic profiling and the ordinate represents log_2_ expression ratio from transcriptomic profiling. Black indicates genes and proteins with no significant differences, red indicates upregulated genes and proteins, orange indicates downregulated genes and proteins, blue indicates upregulated genes but downregulated proteins, purple indicates upregulated genes but proteins with no significant differences, pink indicates genes with no significant differences but downregulated proteins, brown indicates genes with no significant differences but upregulated proteins, green indicates downregulated genes but proteins with no significant differences, and yellow indicates downregulated genes but upregulated proteins.

As the samples in transcriptomics and proteomics analyses were acquired simultaneously, the correlation between mRNA and protein expression profiles was comprehensively investigated. A total of 5,426 genes were detected at both the transcriptional and translational levels (Figure 5A and Supplementary Table S7). The Pearson’s correlation coefficient was 0.6117 (*p* < 0.01) (Figure 5B), indicating a moderate positive correlation between the mRNA and protein level changes. Quantified genes and proteins were categorized into nine groups based on the pattern of changes at the mRNA and protein levels (Figure 5B). In the first quadrant (blue dots), the mRNA level was upregulated, but the protein level was downregulated, including three genes. In the second quadrant (purple dots), the mRNA level was upregulated, but the protein level did not change significantly, including 70 genes. In the third quadrant (red dots), both the mRNA and protein levels of 88 genes were upregulated. In the fourth quadrant (pink dots), the mRNA level did not show a clear change, while the protein level was downregulated, including 33 genes. In the fifth quadrant (black dots), both the mRNA and protein levels of 4,028 genes did not change appreciably. In the sixth quadrant (brown dots), the mRNA level did not show a clear change, while the protein level was upregulated, including 46 genes. In the seventh quadrant (orange dots), both the mRNA and protein levels were synchronously downregulated, including 61 genes. In the eighth quadrant (green dots), the mRNA level was downregulated, but the protein level did not change significantly, including 207 genes. In the ninth quadrant (yellow dots), the mRNA level was downregulated, but the protein level was upregulated, including one gene (Supplementary Table S8). Furthermore, the majority of genes congregated in the center of the coordinate, indicating that these genes’ expression levels were constant at both the transcriptional and translational levels. The data also revealed significantly discordant regulation at the transcriptional and translational levels (i.e., the first, second, fourth, eight, and ninth quadrants). Interestingly, the directions of mRNA and protein changes of four genes were opposing [i.e., apolipoprotein C3 (*Apoc3*), TNF receptor superfamily member 6b (*Tnfrsf6b*), lysyl oxidase like 1 (*Loxl1*), and apolipoprotein A-I (*Apoa1*)]. The latter could be caused by regulation at several levels, such as post-transcriptional processing, degradation of the transcript, translation, post-translational processing, and modification.

### PPI Network of DEPs

**FIGURE 6:**
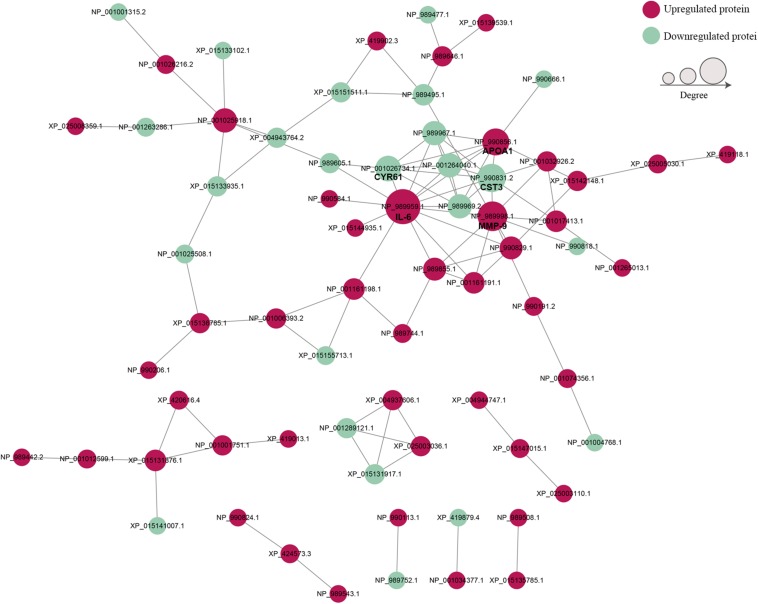
The protein–protein interaction networks of DEPs. Red nodes indicate upregulated proteins and green nodes indicate downregulated proteins. Node size indicates high (large) or low (small) interaction degrees. Network edges represent protein–protein associations.

To establish the interactions of DEPs, the STRING database was used to construct a PPI network (Figure 6). The PPI network consisted of 69 proteins, and the top 5 proteins with higher degree scores were selected as central proteins [i.e., IL-6 (NP_989959.1), MMP-9 (NP_989998.1), cystatin C (CST3, NP_990831.2), APOA1 (NP_990856.1), and cysteine-rich angiogenic inducer 61 (CYR61, NP_001026734.1)] (Supplementary Table S9). Among these proteins, IL-6 had the highest degree of connection and interaction with 14 proteins. These 5 proteins, especially IL-6, may play important roles in the development of acute infectious MS-induced arthritis.

### DEGs and DEPs Involved in MS-Induced Arthritis

To further understand the mechanism of MS-induced arthritis, the top DEGs and DEPs were further filtered. Based on a review of the literature and the GO and KEGG enrichment analyses, the following key proteins and genes related to MS-induced arthritis were identified: extracellular matrix protein 1 (ECM1), transforming growth factor-β2 (TGF-β2), platelet-derived growth factor receptor alpha (PDGFRA), serum amyloid A (SAA), IL-6, IL-8, IL-1β, neuropilin-1 (NRP1), glutamate-ammonia ligase (GLUL), thrombospondin 2 (THBS2), regulator of cell cycle (RGCC), metalloproteinase inhibitor 3 (TIMP3), matrix metalloproteinases (MMPs), nitric oxide synthase 2 (*Nos2*), mitogen-activated protein kinase 11 (*Mapk11*), caspase 8 (*Casp8*), caspase 3 (*Casp3*), apoptosis-inducing factor (*Aifm1*), nuclear factor of kappa light polypeptide gene enhancer in B-cells 1 (*Nf*κ*b1*), htrA serine peptidase 3 (*htrA3*), B-cell CLL/lymphoma 2 (*Bcl2*), *Apoa1*, Complement C1s *(C1s)*, metallothionein 4 (*Mt4*), and *Tlr15* (Table 1).

**TABLE 1:** Critical genes and proteins involved in MS-induced arthritis.

	Gene name	Description	Gene ID	mRNA_log_2_FC	pep_log_2_FC	References
Proliferation-regulated factors	*Ecm1*	Extracellular matrix protein 1	107049123	2.13844959	0.754459974	Mongiat et al., 2003
	*Tgf-*β*2*	Transforming growth factor beta 2	421352	1.069379238	0.740711733	Allen et al., 1990; Elshabrawy et al., 2015
	*Pdgfra*	Platelet-derived growth factor receptor alpha	395509	2.757529736	1.358396262	Watanabe et al., 2002; Madarampalli et al., 2019
	*Saa*	Serum amyloid A	423079	11.92724632	1.599793852	Lee et al., 2006; Hatanaka et al., 2011; Connolly et al., 2016
Apoptosis-related factors	*Nos2*	Nitric oxide synthase 2	395807	3.393184476	NA	Lavric et al., 2007; Lavric et al., 2008; Dusanic et al., 2012
	*Mapk11*	Mitogen-activated protein kinase 11	417739	–1.955189293	NA	Dusanic et al., 2012
	*Casp8*	Caspase 8	395284	0.230119267	0.09085343	
	*Casp3*	Caspase 3	395476	0.062583414	–0.112474729	
	*Aifm1*	Apoptosis-inducing factor	428688	–0.062502318	0.041242982	
	*Nf*κ*b1*	Nuclear factor of kappa light polypeptide gene enhancer in B-cells 1	396033	0.581189669	0.051024003	
	*htrA3*	htrA serine peptidase 3	422868	0.582529237	0.260627908	
	*Bcl2*	B-cell CLL/lymphoma 2	396282	–1.128205565	NA	
Inflammatory mediators	*Il-1*β	Interleukin 1β	395196	10.44244078	0.988412026	Narat et al., 1998; Lavric et al., 2007; Dusanic et al., 2014
	*Il-6*	Interleukin 6	395337	8.876096951	2.033158667	
	*Il-8*	Interleukin 8	396495	9.695048495	2.287767778	Narat et al., 1998; Bodolay et al., 2002; Lavric et al., 2007; Dusanic et al., 2014
Pro-angiogenic factors	*Nrp1*	Neuropilin-1	395560	1.583486767	0.449957484	Kong et al., 2010
	*Glul*	Glutamate-ammonia ligase	396489	4.223126274	2.119024103	Eelen et al., 2018
	*Thbs2*	Thrombospondin 2	414837	1.537056154	0.564622052	Park et al., 2004
Anti-angiogenic factors	*Rgcc*	Regulator of cell cycle	418833	–3.326714915	–1.442222329	An et al., 2009
	*Timp3*	Metallopeptidase inhibitor 3	396483	–4.398041844	–1.02620507	Chen et al., 2014
MMPs	*Mmp-1*	Matrix metallopeptidase 1	418982	7.037356709	NA	Elshabrawy et al., 2015
	*Mmp-2*	Matrix metallopeptidase 2	386583	–1.07696931	–0.279283757	Elshabrawy et al., 2015; Cizelj et al., 2016
	*Mmp-9*	Matrix metallopeptidase 9	395387	5.365580167	0.779049553	Burrage et al., 2006; Elshabrawy et al., 2015
	*Mmp-10*	Matrix metallopeptidase 10	418981	5.831697437	NA	Elshabrawy et al., 2015
	*Mmp-13*	Matrix metallopeptidase 13	395683	–1.282777798	NA	
	*Mmp-27*	Matrix metallopeptidase 27	395850	3.830092934	NA	
Others	*Apoa1*	Apolipoprotein A1	396536	–1.382497329	0.556797247	de Seny et al., 2015
	*C1s*	Complement C1s	418294	1.975691423	0.867896464	Nakagawa et al., 1999
	*Mt4*	Metallothionein 4	396212	3.178141448	0.906890596	Youn et al., 2002
	*Tlr15*	Toll like receptor 15	421219	2.199816288	NA	Oven et al., 2013

*NA, not available.*

### Validation of Gene Expression Levels

**FIGURE 7:**
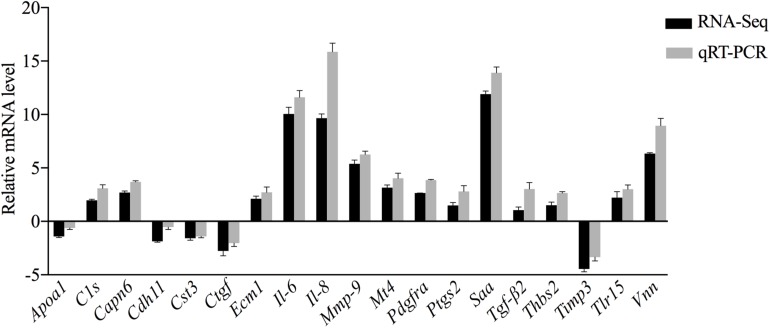
Comparative analysis of the qRT-PCR and transcriptome sequencing results of DEGs in CSF exposed to MS.

To further confirm the transcriptomic and proteomic results, 19 genes were selected for validation by qRT-PCR. Among these selected genes, 14 genes (i.e., *C1s*, *Capn6*, *Ecm1*, *Il-6*, *Il-8*, *Mmp-9*, *Mt4*, *Pdgfra*, *Ptgs2*, *Saa*, *Tgf-*β*2*, *Thbs2*, *Tlr15*, and *Vnn*) were upregulated, while the other 5 genes (i.e., *Apoa1*, *Cdh11*, *Cst3*, *Ctgf*, and *Timp3*) were downregulated. qRT-PCR results exhibited a similar expression tendency as the transcriptomics analysis, confirming the reliability of the transcriptome sequencing results (Figure 7). The trend in the expression levels of *Apoa1* revealed by qRT-PCR was inconsistent with the changes in abundance of its encoded protein detected in the TMT analysis. However, this trend will require further investigation.

## Discussion

MS is a major bacterial pathogen affecting the poultry industry worldwide with infections incurring enormous economic losses (Kreizinger et al., 2017; Sun et al., 2017; Xue et al., 2017; Kursa et al., 2019). Clinical signs of MS infection include infectious synovitis, primarily of the hock joints and foot pads (Kleven et al., 1975). Acute infectious arthritis caused by MS is characterized by damage to the joint cavity. In humans, observations have prompted speculation that synovial fibroblasts (SF) in arthritis are key cells that drive pathological processes (Ospelt et al., 2004; Bartok and Firestein, 2010; Carmona-Rivera et al., 2017; Falconer et al., 2018). SF can release proinflammatory mediators and chemotactic factors that activate complement factors, resulting in inflammation (Bodolay et al., 2002; Mathew and Ravindran, 2014; Yoshitomi, 2019). SF may be proliferative, migratory, and invasive (Falconer et al., 2018). This ability of SF, along with the impairment of host-defense mechanisms, damages the joint cavity and articular cartilage (Mathew and Ravindran, 2014; Yoshitomi, 2019). According to a previous study, one mechanism of acute infectious arthritis may be direct microbial invasion (Mathew and Ravindran, 2014). In the acute phase of infection, live MS is detected in the SF fluids of infected birds, implying that MS may come into direct contact with CCH and CSF (Kerr and Olson, 1970; Dusanic et al., 2012).

A better understanding of host-bacteria interactions would elucidate the molecular mechanism of MS-induced arthritis. The application of high-throughput methods on MS-induced arthritis could lead to the discovery of pathogenesis-related genes and proteins. However, high-throughput methods have not been applied in most studies of host-MS interactions, with the exception of two studies that utilized an avian macrophage microarray/avian innate immunity microarray (Lavric et al., 2008) and a phenotype microarray (Dusanic et al., 2014). To uncover the molecular mechanisms of MS-induced arthritis, primary CSF were prepared by the explanted tissue culture method in this study (Supplementary Figure S1). Then, the DEGs and DEPs in MS-infected CSF were investigated, and critical genes involved in arthritis were identified by transcriptome and proteome analyses.

Recent studies demonstrated that MS-invaded host cells incubated and altered the gene expression of these cells after 24, 48, or 72 h (Dusanic et al., 2009; Buim et al., 2011; Dusanic et al., 2012, 2014; Cizelj et al., 2016). Combining these studies and comprehensive analysis, we employed high-throughput methods to analyze the gene expression of MS-infected CSF 48 hpi. By utilizing DEGs and DEPs and drawing on previous research on arthritis, this study aimed to identify critical genes or proteins involved in arthritis caused by MS infection in chickens. In total, 15,400 genes and 5,537 proteins in MS-treated CSF were identified (Supplementary Tables S3, S5). Of which, 1,137 genes and 129 proteins were upregulated, while the remaining 1,210 genes and 92 proteins were downregulated (Figure 1 and Supplementary Tables S4, S6). For the most part, the correlation analysis of the transcriptome and proteome data revealed that the changes in proteins were consistent with the transcriptome results. Although no difference was detected between the transcriptional and translational regulation of most of the identified genes, four genes opposed this trend (i.e., *Apoc3*, *Tnfrsf6b*, *Loxl1*, and *Apoa1*), which may be affected by post-transcriptional regulation. However, further research is needed to investigate this issue. Additionally, 149 genes (upregulated, *n* = 88; downregulated, *n* = 61) were significantly differentially expressed at both the transcriptional and translational levels in the MS-infected group (Figure 4B and Supplementary Table S7). To further extract related information on MS-induced infectious synovitis and arthritis, 149 genes were analyzed. Although a direct answer does not exist, the relevant literature and GO/KEGG enrichment analyses provide some clues.

A previous study reported hypertrophy and hyperplasia of synovial cells in the joints of MS-infected chickens with fibroplasia and acute inflammatory cells causing the synovial membrane to thicken (Kerr and Olson, 1970). This pathological phenomenon is possibly due to the hyperproliferation and defective apoptosis of SF (Zhang et al., 2009; Wilkinson and Henley, 2010). In the present study, several proteins, including ECM1 (Mongiat et al., 2003), TGF-β2 (Allen et al., 1990), PDGFRA (Watanabe et al., 2002; Madarampalli et al., 2019), and SAA (Lee et al., 2006; Hatanaka et al., 2011), that regulate the proliferation of human arthritis were upregulated at both the mRNA and protein levels. As these genes may also increase the proliferative capacity of CSF, their overexpression may explain CSF hyperplasia, as well as the altered resistance to apoptosis. A previous study demonstrated that apoptosis-induced death of MS-infected CCH was a result of the upregulation of several pro-apoptotic genes, including *Nos2*, *Mapk1*, *Casp8*, *Casp3*, *Aifm1*, *Nf*κ*b1*, *htrA3*, and *Bcl2* (Dusanic et al., 2012). However, with the exception of *Nos2*, the transcription of these genes in CSF was unchanged or downregulated in this study. Dysregulation of apoptosis and the proliferation of CSF due to multiple genes may play roles in this hyperplasia.

Previous studies reported human SF-mediated inflammation and autoimmunity via the production of inflammatory mediators in rheumatoid arthritis (RA) (McInnes and Schett, 2011; Bustamante et al., 2017). In the present study, the expression of various inflammatory mediators, including *Il-6*, *Il-8*, *Il-1*β, and *Nos2*, increased in MS-infected CSF, which matches the findings of previous studies on MS. Lavric et al. (2007, 2008) found that viable MS and MS proteins stimulated chicken monocyte-derived macrophages and HD11, and CCH secreted IL-6, IL-8, and IL-1β. In a previous study that utilized phenotype microarrays to investigate the effects of MS on the global metabolic activity of CCH, MS-infected CCH were found to be sensitive to IL-6, IL-1β, IL-8, TNF-α, and IFN-γ (Dusanic et al., 2014). Previous studies also found that human SF secreted various cytokines and that cytokine networks at inflammatory sites largely contributed to the pathogenesis of arthritis and the perpetuation of inflammation (Bartok and Firestein, 2010; Emery, 2012; Kihara et al., 2017). Exposure to these cytokines led to chrondrocyte deprivation in cartilage over time (McInnes and Schett, 2011). Another study found that through the secretion of cytokines, human SF played a role in the persistence of inflammation in the synovium via the recruitment and retention of effector cells in the immune system (Ritchlin, 2000). Thus, it is likely that proinflammatory cytokines upregulated in CSF play a role in the pathogenesis of MS-induced arthritis.

In addition to the production of inflammatory cytokines, another characteristic of synovium inflammation in RA is angiogenesis, which appears to be necessary for pannus development (MacDonald et al., 2018). The expansive synovial tissue, “pannus,” which is found at the cartilage-bone interface, cloaks the cartilage and erodes it into bone (Bartok and Firestein, 2010). The ability of RASF to secrete a number of proangiogenic factors suggests that RASF also contributes to blood vessel growth, which is necessary for sustaining pannus formation and the arthritis process (Mor et al., 2005). An investigation conducted by Kerr and Olson (1970) observed the formation of granulomatous pannus in the medullary spaces of chickens inoculated experimentally or contact-infected with MS. The present study demonstrated that several proangiogenic factors were upregulated, including ECM1 (Mongiat et al., 2003), IL-8 (Bodolay et al., 2002), TGF-β2 (Elshabrawy et al., 2015), SAA (Connolly et al., 2016), MMP-9 (Burrage et al., 2006), NRP1 (Kong et al., 2010), GLUL (Eelen et al., 2018), and THBS2 (Park et al., 2004), while antiangiogenic factors, including RGCC (An et al., 2009), as well as TIMP3 (Chen et al., 2014), were downregulated at both the mRNA and protein levels. These findings suggest that MS-infected CSF may contribute to blood vessel growth, which is necessary for sustaining pannus formation and the arthritis process.

The growth of new blood vessels requires the breakdown of the surrounding ECM. Principal enzymes involved in this process include MMPs (Chen et al., 2014), as well as a family of zinc-containing, calcium-dependent proteinases that degrade major components of ECM (Murphy et al., 2002). Although collagenases (i.e., MMP-1, -8, and -13), gelatinases A and B (i.e., MMP-2 and -9), stromelysins (i.e., MMP-3, -10, and -11), and matrilysins (i.e., MMP-7 and -26) are expressed at low levels in normal human joint tissues, they are highly expressed in arthritic joints (Elshabrawy et al., 2015). In RA, cartilage destruction depends on the balance between MMPs and TIMPs. When the balance favors MMPs, cartilage degradation proceeds (Yoshitomi, 2019). In this study, *Mmp-1*, *-9*, *-10*, and *-27* were upregulated, while *Mmp-2* and *-13* were downregulated at the mRNA level. The MMP-9 protein was upregulated and TIMP was downregulated at the protein level. These findings suggest that CSF may contribute to MS-induced arthritis via MMPs.

Additionally, the expression of certain genes changed at the mRNA and/or protein levels, including *Apoa1* (de Seny et al., 2015), *C1s* (Nakagawa et al., 1999), *Mt4* (Youn et al., 2002), and *Tlr15* (Oven et al., 2013). A review of the literature revealed that these genes may participate in the pathogenesis of MS-induced infectious synovitis in chickens.

## Conclusion

In this study, the gene and protein expression changes caused by MS strain HN01 infection in CSF were investigated. Several critical DEGs and DEPs involved in MS-induced arthritis were identified. These genes and their protein products in infected CSF consisted of proliferation- and apoptosis-related factors, inflammatory mediators, proangiogenic factors, antiangiogenic factors, and MMPs, as well as other arthritis-related proteins, indicating that these genes may play important roles in MS-induced arthritis. The integrated analysis of transcriptome and proteome data advances our understanding of the interplay between CSF and MS. However, future work is needed to elucidate the interactions observed therein.

## Data Availability Statement

The datasets generated for this study can be found in the NCBI Sequence Read Archive: SRR10479009, SRR10479010, SRR10479011, SRR10479012, SRR10479013, and SRR10479014. ProteomeXchange Consortium: PXD016513.

## Ethics Statement

The animal experiment was carried out in Jiangsu Academy of Agricultural Sciences with the approval of the Committee on the Ethics of Animal Experiments of (JAAS No. 20141107). The protocol conformed to the guidelines of Jiangsu Province Animal Regulations (Government Decree No. 45) in accordance with international law.

## Author Contributions

RL conceived and designed the experiments, carried out the experiments, analyzed the data, and wrote the manuscript. BX participated in performing experiments. SY participated in revising the manuscript. JZ, HS, CL, FL, and QP participated in analyzing and interpreting the data. XZ supervised and guided this work. All the authors have read and approved the final version of the manuscript.

## Conflict of Interest

The authors declare that the research was conducted in the absence of any commercial or financial relationships that could be construed as a potential conflict of interest.

## Footnotes

1 https://www.ncbi.nlm.nih.gov/genome/111?genome_assembly_id=451987

2 http://www.ebi.ac.uk/GOA/

3 http://www.ebi.ac.uk/interpro/

4 http://www.genome.jp/kaas-bin/kaas_main

5 http://string-db.org/
