# Supplementary figures and images for: Integrated Transcriptomic and Proteomic Analyses of the Interaction Between Chicken Synovial Fibroblasts and Mycoplasma synoviae
Source: Front Microbiol. 2020 Apr 3;11:576. doi: 10.3389/fmicb.2020.00576 (PMC7147270; doi:10.3389/fmicb.2020.00576)

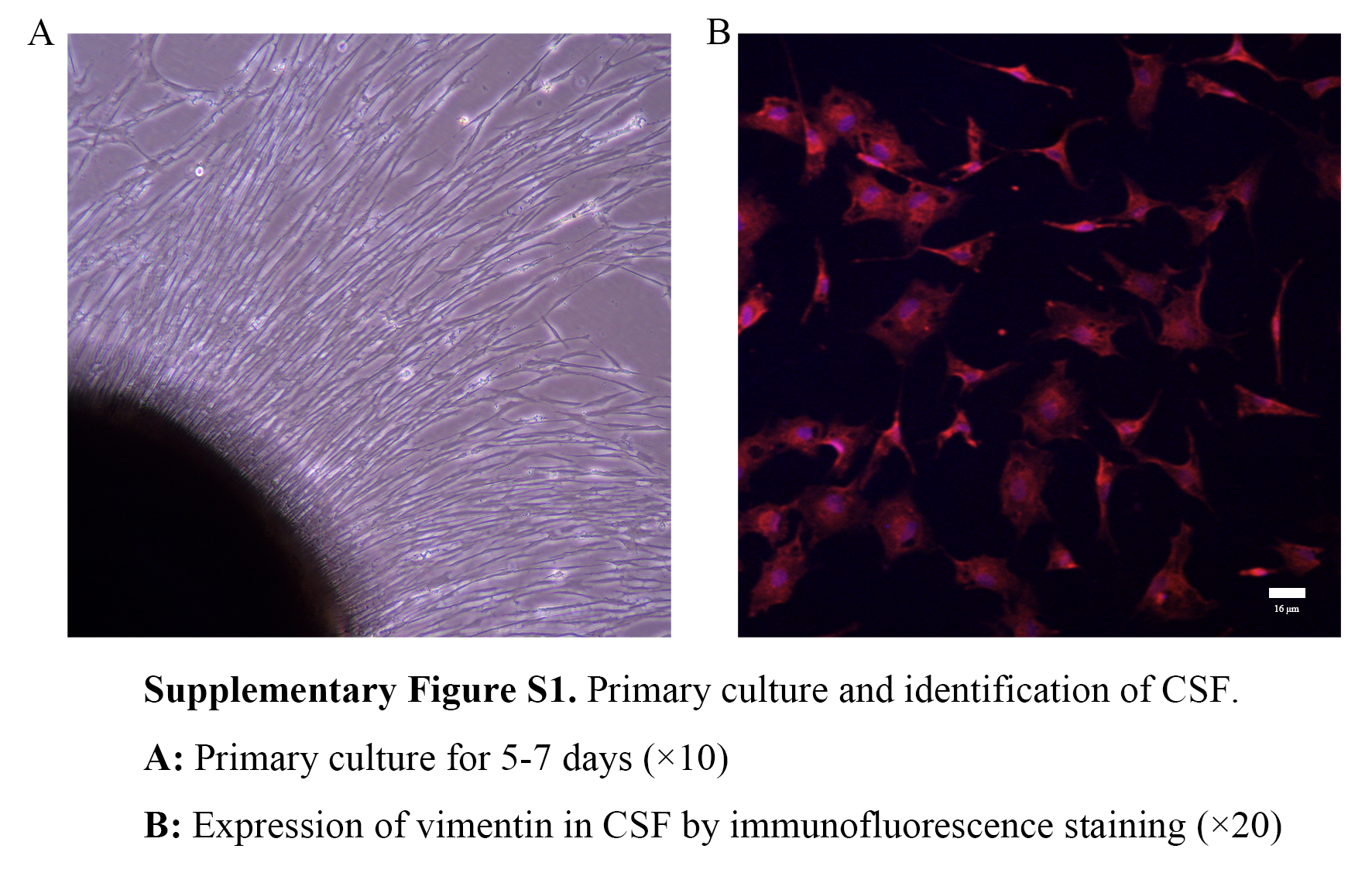

Supplement: Supplementary file 1 [file Image_1.TIF]
